# Actionability and Simulation: No Representation without Communication

**DOI:** 10.3389/fpsyg.2016.01457

**Published:** 2016-09-26

**Authors:** Jerome A. Feldman

**Affiliations:** International Computer Science Institute, University of California, Berkeley, BerkeleyCA, USA

**Keywords:** actionability, connectionist, fitness, neural code, representation, simulation

## Abstract

There remains considerable controversy about how the brain operates. This review focuses on brain activity rather than just structure and on concepts of action and actionability rather than truth conditions. Neural Communication is reviewed as a crucial aspect of neural encoding. Consequently, logical inference is superseded by neural simulation. Some remaining mysteries are discussed.

## Introduction

This Frontiers project on “Representation in the Brain” is extremely timely. Despite significant theoretical and experimental advances, there is still considerable confusion on the topic. Wikipedia says: *Representation:* “A **mental representation** (or **cognitive representation**), in philosophy of mind, cognitive psychology, neuroscience, and cognitive science,” is a hypothetical internal cognitive symbol that represents external reality, or else a mental process that makes use of such a symbol: a formal system for making explicit certain entities or types of information, together with a specification of how the system does this. “https://en.wikipedia.org/wiki/Mental_representation, August/8/2016.”

The definition above presupposes a separation between data and process that is true of books and computers but is utterly false in neural systems. In this article we use the term “encoding” instead of “representation”. The brain is not a set of areas that represent things, but rather a network of circuits that do things. It is the activity of the brain, not just its structure, that matters. This immediately brings focus on actions and thus circuits. This paper will not attempt to describe (the myriad) particular brain circuits but will focus on the mechanisms for coordination among the local information transfer and areas and circuits missing in most discussions of “representation.”

**FIGURE 1:**
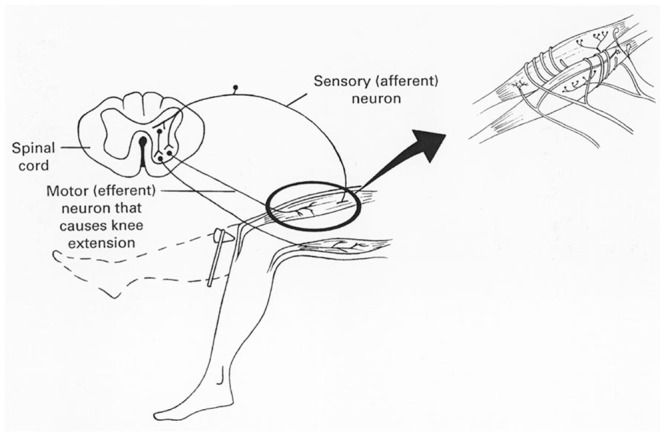
**Knee-jerk Reflex Circuit**.

For concreteness, let’s start with a simple, well-known, neural circuit, the knee-jerk reflex shown in **Figure 1.** We are mainly concerned with the simplicity of this circuit; there is a single connection in the spinal cord that converts sensory input to action. The knee-jerk reflex is behaviorally important for correcting a potential stumble while walking upright. The doctor’s tap reduces tension in the upper leg muscle and this is detected by stretch receptor in the muscle spindle, sending neural spike signals to the spinal cord. The downward spike signals directly cause the muscle to contract and the leg to “jerk.” Not shown here are the many other circuit connections that support coordination of the two legs, voluntary leg jerking, etc.

There are several general lessons to be learned from this simple example. Essentially everyone now agrees that neurons are the foundation of encoding knowledge in the brain. But, as the example above shows, it is the *activity* of neurons, not just their connections, that supports the functionality.

The example involved motor activity, but the basic point is equally valid for perception, thought, and language, they are all based on neural activity. There are three essential considerations in discussing neural circuits – the computational properties of individual neurons, the structure of networks and the communication mechanisms involved.

Of these three, it is communication mechanisms that have been studied the least and this fact is the basis for the subtitle “no representation without communication”. “Neural Communication and Representation,” below is a brief review of what has been called the neural code (Feldman, 2010a). Considerations from neural computation also constrain possible answers to traditional questions like localist vs. distributed representations. Actionability and Simulation goes further and directly addresses the consequences of accepting action and actionability as the core brain function that needs to be explained. The final Conclusions section also considers remaining unsolved mysteries involving the mind-brain problem, some of which are ubiquitous in everyday experience

## Neural Communication and Representation

One key question concerns the basic mechanisms of neural communication. It is now accepted that the dominant method is transmission of voltage spikes along axons and through synapses that are connections to downstream neural processes. Neural spikes are an evolutionary ancient development that remains nature’s main technique for fast long distance information passing (Meech and Mackie, 2007). Other neural communication mechanisms are either extremely local (e.g., gap junctions) or much slower (e.g., hormones). Neural spikes serve a wide range of functions.

Much of the chemistry underlying neural spikes goes back even earlier (Katz, 2007; Meech and Mackie, 2007). The earliest use of spiking neurons is to signal coordinated action as in the swimming of jellyfish. This kind of direct action remains one of the main functions of neural spikes as suggested by **Figure 1.** Due to the common chemistry, all neural spikes are of the same duration and size (Katz, 2007; Meech and Mackie, 2007). The basic method of neural information transfer is direct –the information depends on which neurons are linked. Most of the information sent by a sensory neural spike train is based on the sending unit. For output, the result of motor control signaling is largely determined by which fibers are targeted. The other variable is timing; there is a wide range of variation in the firing rate and conduction time of neural spikes.

The other factor on neural computation is resource limitations (Lennie, 2003). The most obvious resource constraint for neural action/decision is time. Many actions need to be fast even at the expense of some accuracy. Some neural systems evolved to meet remarkable timing constraints. Bats and owls make distinctions that correspond to timing differences at the 10 μs level -much faster than neural response times. A second key resource is energy; neural firing is metabolically costly (Lennie, 2003) and brains evolved to conserve energy while meeting performance requirements. The three factors of accuracy, timing, and resources are the elements of a function that conditions neural computation.

We can show why it is not feasible for one neuron to send an abstract symbol (as in ASCII code) to another as a spike pattern (Feldman, 1988). It is known experimentally that the firing of sensory (e.g., visual) neurons is a function of multiple variables, often intensity, position, velocity, orientation, color, etc. It would take an extremely long message to transmit all this as an ASCII like code and neural firing rates are too slow for this, even omitting the stochastic nature of neural spikes. Even if such a message were somehow encoded and transmitted downstream, it would require a complex computation to decode it and combine the result with the symbolic messages of neighboring cells and then build a new symbolic message for the further levels. Language is a symbolic system that is processed by the brain, but nothing at all like abstract symbols occurs at the individual unit level.

In the past, there have been debates about whether neural representations were basically punctuate with a “grandmother cell” (Bowers, 2009) for each concept of interest. The alternative was basically holographic (with each item encoded by a pattern involving all the units in a large population). It has been understood for decades (Feldman, 1988) that neither extreme could be realized in the neural systems of nature.

Having just a single unit coding the element of interest (concept) is impractical for many reasons. The clearest is that the known death of cells would cause concepts to vanish. Also, the firing of individual units is probabilistic and would not be a stable representation. It is easy to see that there are not nearly enough units in the brain to capture all the possible combinations of sizes, motions, shapes, colors, etc., that we recognize, let alone all the non-visual concepts. The grandmother cell story was always a straw man— using a modest number (∼10) units per concept could overcome all these difficulties.

The holographic alternative was originally more popular because it used the techniques of statistical mechanics. But it is equally implausible. This is easy to see informally and was proved as early as (Willshaw et al., 1969). Suppose a system should represent a collection of concepts (e.g., words) as a pattern of activity over some number *M* (say 10,000,000) neurons. The key problem is cross-talk: if multiple words are simultaneously active, how can the system avoid interference among their respective patterns. Willshaw et al. (1969) showed that the best solution is to have each concept represented by the activity of only about logM units, which would be about 24 neurons in our example. There are many other computational problems with holographic models (Feldman, 1988). For example if a concept required a pattern over all *M* units, how would that concept combine with other concepts without cross-talk. Even more basically, there is no way that a holographic representation could be transmitted from one brain circuit to another.

There is a wide range of converging experimental evidence (Quiroga et al., 2008; Bowers, 2009) showing that neural encoding relies on a modest number (10–100) of units. There is also some overlap—the same unit can be involved in the representation of different items. For several reasons, not all of them technical, some papers continue to refer to these structured representations as “sparse population codes.” A much more appropriate term would be redundant circuits.

There is now a general consensus on the basis of neural spike signaling and encoding. There are a number of specialized neural structures involving delicate timing. The relative time of spike arrival is also important for plasticity. But the main mechanism for neural signaling is frequency encoding in functional circuits of low redundancy.

## Actionability and Simulation

Given that knowledge is encoded in the brain as active circuits, the next big question concerns the nature of this embodied knowledge. The key idea is that living things and their brains evolved to **act** in the physical and social world. Action is evolutionarily much older than symbolic thought, belief, etc., and is also developmentally much earlier in people. Sensory actions loops like the knee-jerk reflex (**Figure 1**) significantly pre-date neurons and are crucial even for single celled animals such as amoeba (Katz, 2007). Only living things act (in our sense); natural forces, mechanisms, etc. are said to act by metaphorical extension (Lakoff and Johnson, 1980).

Fitness is the technical term for nature’s assessment of agents’ actions in context. Natural selection assures that creatures with sufficiently bad choices of actions do not survive and reproduce. The term ***actionability*** has been defined as an organism’s internal assessment of its available actions in context (Feldman and Narayanan, 2014). Of course, such an internal calculation will rarely be optimal for fitness, but evolution selects systems where the match is good enough.

Actions, in this formulation, include persistent change of internal state: learning, memory, world models, self-concept, etc. In animals, perception is best-fit, active, and utility/affordance based (Parker and Newsome, 1998). The external world (e.g., other agents) is not static so internal models need **simulation**. Simulation involves imagining actions and estimating their likely consequences before actually entailing the risks of trying them in the real world (Bergen, 2013). Both actionability assessment and simulation rely on good (but not veridical) internal models. This is another fundamental property of neural encoding.

Another important issue concerns the roles of **rules**, including logical rules in the brain. Once a simulation has been done successfully, people can cache (remember) the result as a rule and thus shortcut a costly simulation. Search in a symbolic model can be viewed as a form of simulation. Learning generalizations of symbolic rules is a crucial process and not well understood.

Communication is an important form of action and is needed for cooperation, as discussed in Neural Communication and Representation. Even single-celled animals, like some amoebas, rely on pheromones for survival, particularly for organizing into stable structures in times of environmental stress (Shorey, 2013). Higher plants and animals rely on communication actions for many life functions. And, of course, language is a characterizing trait of people. Much of what we know and what we need to learn about “representation in the brain” is concerned with language.

Actionability, not non-tautological truth, is what an agent/animal can actually compute. We have no privileged access to external truth or to our own internal state. This entails the **operationality** of all living things. In science, operationalism states that theories should be evaluated for their explanatory and predictive power, not as assertions of the reality of their terms, e.g., electrons. Living things incorporate structures that model the external and internal milieus to enhance fitness. Evolution constrains these structures to be consistent with reality.

The basic actionability story applies to all living things, but there are profound differences between different species. One crucial divide/cline is ***volitional*** action and communication – the boundary is not clear, but birds are above the line; protozoans, plants below. We assume that, in nature, neurons are necessary for volition (Damasio, 1999). Volitional actions have automatic components and influence, e.g., speech. For example, deciding to talk is volitional; the details of articulation are automatic.

Learning is obviously a foundation of intelligent activity and also important in much simpler organisms. The current revolution in big data, deep learning, etc., can help provide insights for this enterprise as well as many others, but is not a model for the mechanisms under study. Structure learning remains to be understood. Observational learning without a model is influenced by the observer’s ability to act in the situation (Iani et al., 2013). In Nature, there is no evidence for tabula-rasa learning and massive evidence against it.

Language is a hallmark of human intelligence and its representation in the brain is of major importance. From our actionability perspective, the crucial question is the neural encoding of ***meaning.*** A tradition dating literally back to the Greeks identifies meaning with “truth” as defined in formal logics. This historical fact wouldn’t matter except that the same definition of meaning dominates much current work in formal linguistics, philosophy, and computer science. But action is evolutionarily much older than symbolic thought, belief, etc., and is also developmentally much earlier in people.

Decades of inter-disciplinary work suggests that the definition of meaning should be expressed in an action-oriented formalism (Narayanan, 1999) that maps directly to embodied mechanisms (Feldman, 2005). For example, the meaning of a word like “push” is captured formally as an action schema that captures the preconditions and resources needed for the action as well as the possible results of the action. Furthermore, all actions inherit from a common control schema (Narayanan, 1999) that models general aspects of action including completion, interrupts, repetition, etc. This action formalism is multi-modal: describing execution, recognition, and planning as well as language.

In addition, the meaning of a word like “push” is assumed to engage neural circuits that produce pushing behavior in people and other animals. There are wide ranging findings that indeed words and images about actions do activate much of the same circuitry as carrying out the action (Garagnani and Pulvermüller, 2016). This is strong evidence about the encoding of actions, action images, and action language in the brain. A further extension of actionability theory accounts for the meaning of metaphorical meanings of words like push in examples like “push for a promotion” (Lakoff and Johnson, 1980). Metaphorical mappings are modeled as mappings from some target domain (here, employment) to an embodied source domain. A remarkable range of phenomena are explained by this theory and, again, there is strong neural support for the connection (Bergen, 2013).

This brings us back to simulation, which was discussed earlier as being necessary for modeling the response of external environment (including other agents) to one’s actions. Some automatic simulations (like dreams) are well understood in mammals, but people rely upon volitional (intentional, purposeful) simulation for many functions including planning and language (Feldman, 2005). Some remarkable new experiments (Pfeiffer and Foster, 2013) suggest that rodents might exhibit volitional simulation, but this remains controversial.

More generally, simulation is a cornerstone of an extensive effort on language theory, embodiment, and application. Volitional simulation has been proposed as the mechanism of planning, mind-reading, etc. (Bergen, 2013). With an appropriate formalism, simulation can yield both causal and predictive inferences (Pearl, 2000). Results of simulations can be cached (remembered) and generalized as rules. The NTL theory of language and thought entails additional mechanisms including construction grammar, mental spaces, mappings, etc. (Feldman, 2010b).

## Conclusions and Mysteries

This Frontiers project on “Representation in the Brain” is extremely timely; despite recent theoretical and experimental advances, there is still considerable confusion on the topic. As is often the case, part of the problem arises from the use of anachronistic terms like “representation” to describe neural computation. There are also surviving revivals of old theories (like holographic memory and field theory) that are incompatible with current findings. But for the most part, there is a good scientific consensus on what could be called a standard theory of neural computation (Parker and Newsome, 1998). This is based on the activity of individual neurons that participate in multiple complex circuits and communicate primarily through spikes transmitted through axons to synapses with processes of downstream cells.

In addition to our improved understanding of the computational primitives of the brain, there are promising advances on theories and experiments at the functional level. The ancient idea that meaning should be equated with logical truth is being replaced by theories that emphasize the function of brains in interacting with the physical and social environments (Kahneman, 2011). In a related development, the idea of language and thought as logical deduction is giving way to theory and experiment grounded in bodily experience and simulation (Bergen, 2013).

However, there are fundamental questions on neural computation that remain mysteries in that there is no plausible theory to account for them. The general mind-body problem is known to be intractable and currently mysterious (Chalmers, 1996). This is one of many deep problems, including quantum phenomena, etc., that are universally agreed to be beyond the current purview of science. But all of these famous unsolved problems are either remote from everyday experience (complementarity, dark matter) or are hard to even define sharply (consciousness, free will, etc.).

There are also problematic ordinary behaviors–recent work (Feldman, 2016) describes some obvious problems in vision that arise every time that we open our eyes and yet are demonstrably **incompatible** with current theories of neural computation, including those presented in this article. The focus was on two related phenomena, known as the *neural binding problem* and the *illusion of a stable visual world*. I, among many others, have struggled with these issues for more than 50 years and I now believe that they are both unsolvable within current neuroscience. By considering some basic facts about how the brain processes image input, (Feldman, 2016) shows that there are not nearly enough brain neurons to compute what we experience as vision. We imagine that we perceive an entire scene at full resolution, but only about 1 degree in the fovea is encoded that precisely. However, the area of visual cortex that encodes the fovea is much too large to be replicated ∼400 times to fully encode a full scene in detail.

I suggest that these facts should induce humility about the prospects for our current neuroscience to yield a complete reductionist account of even concrete aspects of vision and other thought processes. So, “representation in the brain” remains one of the central scientific questions of our time, if not of all time.

## Author Contributions

The author confirms being the sole contributor of this work and approved it for publication.

## Conflict of Interest Statement

The author declares that the research was conducted in the absence of any commercial or financial relationships that could be construed as a potential conflict of interest.
